# CLINICAL TRIAL OF METFORMIN FOR FRAILTY PREVENTION IN COMMUNITY-DWELLING OLDER ADULTS WITH PRE-DIABETES

**DOI:** 10.1093/geroni/igac059.2117

**Published:** 2022-12-20

**Authors:** Sara Espinoza, Chen-pin Wang, Joel Michalek, Daniel MacCarthy, Tiffany Cortes, Becky Powers, Nicolas Musi

**Affiliations:** University of Texas Health Science Center at San Antonio, San Antonio, Texas, United States; University of Texas Health Science Center at San Antonio, San Antonio, Texas, United States; University of Texas Health Science Center at San Antonio, San Antonio, Texas, United States; University of Texas Health Science Center at San Antonio, San Antonio, Texas, United States; University of Texas Health Science Center at San Antonio, San Antonio, Texas, United States; University of Texas Health Science Center at San Antonio, San Antonio, Texas, United States; University of Texas Health Science Center at San Antonio, San Antonio, Texas, United States

## Abstract

**Background:**

Metformin may reduce frailty through improving insulin resistance and inflammation, aging mechanisms known to increase frailty risk. We describe a randomized clinical trial of metformin for frailty prevention in community-dwelling older adults with pre-diabetes and provide baseline characteristics of randomized participants.

**Methods:**

Older adults (65+ years) are studied in this randomized, double-blind, placebo-controlled trial of metformin (max 2,000 mg/day). Pre-diabetes, required for inclusion, is assessed by 2-hour oral glucose tolerance test (OGTT). Individuals with glomerular filtration rate < 45 mL/min and frail individuals (Fried criteria) are excluded. The primary outcome, frailty, is assessed by both Fried criteria and frailty index. Secondary outcomes are physical function (short physical performance battery), lower extremity strength (Biodex), 6-minute walk, inflammation (systemic and skeletal muscle tissue), muscle insulin signaling, insulin sensitivity (insulin clamp), glucose tolerance (OGTT), and body composition (dual-energy x-ray absorptiometry). Subjects are followed for 2 years with safety assessments every 3 months and frailty assessment and OGTT every 6 months.

**Results:**

145 participants (49% female, 35% Hispanic) are randomized. Mean age is 71.8 ± 5.4 years (range: 65-88), body mass index is 30.8 ±6 kg/m2, and Hemoglobin A1c is 5.6 ±0.4%. Using Fried criteria, 63.4% have frailty score of 0, 30.3% a score of 1, and 6.2% a score of 2.

**Conclusion:**

Metformin is being examined as a potential therapeutic agent to prevent frailty in older adults with pre-diabetes. Findings from this trial may have future implications for screening and treatment of pre-diabetes in older adults for the prevention of frailty.

